# Tumors of the Pancreatic Body and Tail

**DOI:** 10.4021/wjon2010.04.200w

**Published:** 2010-04-30

**Authors:** Savio George Barreto, Parul J. Shukla, Shailesh V. Shrikhande

**Affiliations:** a Department of General and Digestive Surgery, Flinders Medical Centre, Adelaide – South Australia; b Department of Gastrointestinal Surgical Oncology, Tata Memorial Hospital, Mumbai, India

**Keywords:** Pancreatic tumor, Neuroendocrine, Cystic, Venous, Arterial

## Abstract

Tumors of the pancreatic body and tail are uncommon. They have a propensity to present late and often attain a large size with local invasion before they produce any clinical symptoms. The current review aims at comprehensively analysing these tumors with respect to their pathology, presentation, the investigation of these tumors, and finally the latest trends in their surgical and medical management.

## Introduction

Tumors of the body and tail of the pancreas constitute one-third of the pancreatic neoplasms. They have always been associated with a poor prognosis due to the late presentation, and hence, advanced stage of the disease at diagnosis. However, this trend is gradually on the decline with the awareness of the existence of these lesions, better radiologic imaging modalities for diagnosis, and the more aggressive treatment strategies adopted in these patients. This review, which will visit the entire spectrum of tumors that can present in the pancreatic body and tail, will also attempt to provide current strategies for the diagnosis and treatment of these uncommon yet, often, surgically challenging tumors.

## Pathology

Tumors in the body and tail include the entire spectrum of exocrine and endocrine neoplasms of the pancreas. Table 1 shows a classification of these lesions. The pathological features of the most common neoplasms are discussed below:

**Table 1:** Classification of pancreatic tumors occurring in the body and tail

Exocrine
Malignant
duct cell carcinoma (90% of all cases)
acinar cell carcinoma
papillary mucinous carcinoma
signet ring carcinoma
adenosquamous carcinoma
undifferentiated carcinoma
mucinous carcinoma
giant cell carcinoma
mixed type (ductal-endocrine or acinar-endocrine)
small cell carcinoma
cystadenocarcinoma (serous and mucinous types)
unclassified
pancreatoblastoma
papillary-cystic neoplasm (this tumor has lower malignant potential, and may be cured with surgery alone)
Borderline
mucinous cystic tumor with dysplasia
intraductal papillary mucinous tumor with dysplasia
pseudopapillary solid tumor

Endocrine
Functioning Tumors
Insulinoma
Gastrinoma
VIPoma
Ghrelinoma
Somatostatinoma
Glucagonoma
GRFoma
PPoma
Carcinoids
Nonfunctioning Tumors

### Ductal adenocarcinoma

Approximately 15% of these tumors are found in the body and tail. They grossly appear as white-yellow, poorly defined, firm masses often obstructing the main pancreatic duct. Microscopic appearance is that of infiltrative glands of various shapes and sizes surrounded by an intense desmoplastic response, extending beyond the grossly defined mass [1]. The nuclei of the cells show marked pleiomorphism, hyperchromasia, loss of polarity, and prominent nucleolei. The epithelial cells often contain mucin and may form papillae and cribriform structures. These tumors frequently invade the vascular, lymphatic, and perineural spaces.

### Cystic neoplasms

The four main classes of cystic pancreatic tumors include the serous cystic, mucinous cystic, intraductal papillary mucinous, and the unusual neoplasms.

Serous cystadenomas (SCAs) vary in size from 6 cm to 10 cm although cysts of even 25 cm have been reported. They are well demarcated and are lined by simple, glycogen–rich cuboidal epithelium and characterized by dense, lace-like, honeycombed matrix of fibrous septae. They have been referred to as microcystic since they are made up of clusters of cysts that are filled with clear watery, non-mucinous and occasionally bloody fluid.

Mucinous neoplasms are made up of cysts that are larger in size than the serous neoplasias and are usually up to 25 cm. The cyst contains, mucinous, viscid fluid. The main features that help in distinguishing it from other cystic neoplasias include the presence of a dense mesenchymal ovarian-like stroma, and the lack of communcation with the main pancreatic ductal system [2, 3]. The Mayo Clinic has divided these tumors for purposes of treatment into 3 subgroups [4]: a) Mucinous cystadenomas (MCA’s) (65 %); b) Non-invasive proliferative MCNs (30 %), with and without dysplasia; and c) Mucinous cyastadenocarcinomas.

Intraductal Papillary mucinous neoplasms (IPMN) are characterized by papillae (intestinal, hepatobiliary, gastric, or rarely, oncocytic) [5-8] arising from the intraductal proliferation of neoplastic mucinous cells. They are associated with the dilatation of the pancreatic duct and / or the ductal side branches that contain mucin. They have been seen to possess 4 predominant patterns [9]: 1) Diffuse main pancreatic duct ectasia; 2) Segmental main pancreatic duct ectasia; 3) Side-branch duct ectasia (“branch-duct” type); 4) Unifocal and multifocal cysts with pancreatic duct communication.

Solid and cystic papillary and epithelial neoplasias (SPEN) have a variegated appearance with solid, cystic, and papillary areas with foci of necrosis and haemorrhages. The degenerative areas have been attributed to vascular ischaemia [10]. The most useful markers are alpha-1-1antitrypsin, alpha-1-antichymotrypsin, neuron specific enolase (NSE), and Vimentin [11].

### Endocrine tumors [12]

1) Insulinoma. Grossly, 40% of these tumors are < 1 cm, and 66% are < 1.5 cm [13]. They present as encapsulated, firm, brown, nodules that are histologically composed of cords and nests of well-differentiated β cells that do not differ from the normal islet cells. 2) Gastrinoma. The tumor size in the pancreas is usually above 2 cm and in the duodenum below 1cm, often very tiny and often multicentric. More than 85% are identifiable in an anatomical triangle bordered by cystic duct, the junctions of the second and third portions of the duodenum, and the junction of the neck and body of the pancreas that has been referred to as the ‘Gastrinoma Triangle’ [14]. 3) Glucagonoma. The histology of the tumor is similar to the entire group of neuroendocrine tumors with the basic difference being the production and release of large quantities of glucagons. 4) VIPoma, PPoma, etc, share the same histology except for the basic difference of the hormone produced.

The aggressiveness of the tumor is defined not by the histology but rather by the behavior.

## Genetics

A summary of the genetic changes encountered in ductal adenocarcinomas, endocrine tumors, and intraductal papillary mucinous neoplasms are listed in Table 2 [15].

**Table 2:** Summary of the genetic alterations in pancreatic tumors according to histopathological type

Adenocarcinoma	Pancreatic endocrine tumors	Intraductal papillary mucinous tumors
*Sporadic*		
K – ras	Men-1	K-ras
P53	P16	P53
P16	P27	P16
DPC4	Cyclin D	
	DPC4	
*Hereditary*		
PRSS1	Men-1	
FAMM (p16)	Von Hippel Lindau (VHL)	
STK11/LKB1	Von Recklinghausen’s	
BRCA 2	disease (NF-1)	
HNPCC	Tuberous Sclerosis	
Li Fraumeni Syndrome (p53)	(TSC1, TSC2)	

## Clinical presentation

Tumors of the body and tail, in general, tend to present late until they produce a clinically discernible swelling. By this time the tumor is usually infiltrating adjacent organs or vascular structures and possibly metastasized via lymphatics to locoregional lymph nodes, or by haematogenous dissemination to distant organs [16, 17]. The difference in the time to detection as compared to the tumors in the head is due to the lack of obstructive symptoms of the biliary and gastric systems. The functioning neuroendocrine tumors, with their characteristic symptom complexes, can be detected provided the clinician is quick to recognize these features.

The most common symptoms encountered are pain (epigastric, and radiating to the back, in case of celiac plexus involvement), weight loss, and new onset diabetes mellitus (especially in patients > 60 yrs). The commonly encountered nonspecific symptoms include anorexia, loss of appetite, weakness and lethargy.

The presence of a palpable tumor inadvertently points to a diagnosis of cystadenoma (esp. mucinous carcinomas) [18] or islet cell tumor, as adenocarcinoma is rarely palpable prior to demise [19].

Cystic tumors of the pancreas, when symptomatic (approx. 25 - 60%) [9, 20], produce pressure symptoms. These are more commonly seen in mucinous tumors with the incidence of symptoms correlating with the risk of malignancy. Abdominal pain weight loss and diarrhoea are common [21]. The less common symptoms are constipation, diarrhoea, abdominal distension, fatigue, early satiety, and in the rare event of functioning tumors, the patient may show signs of hypoglycemia [22]. Hemorrhagic complications secondary to gastric involvement, portal hypertension, haemobilia, or haemosuccus pancreaticus, can be seen in malignant mucinous neoplasms [23, 24]. In rare cases of SPEN, patients have presented with acute abdominal pain due to rupture of the tumor [25]. IPMNs, when symptomatic, present with signs of chronic pancreatitis and pancreatic exocrine insufficiency, i.e. pain, steatorrhoea, weight loss, urgency, diabetes, etc. They can also present as acute or recurrent pancreatitis. The main distinguishing feature is the lack of aetiology for acute pancreatitis on obtaining a history in such a patient. Diabetes is found to be associated with mucinous tumors especially those that are malignant [26]. Rare associations with Peutz-Jeghers and Zollinger Ellison syndrome have been described [27, 28].

Endocrine tumors present with characteristic syndrome complexes. Insulinomas are characterised by the Whipples triad that includes symptoms of hypoglycemia accompanied by plasma glucose levels < 3.0 mmol/L relieved by glucose administration [29]. Gastrinomas lead to symptoms of peptic ulcer disease (90%) often not responsive to acid suppressive therapy or associated with relapse despite therapeutic compliance [30]. Glucagonomas present with a rash described as dermatitis necrolysis migrans, anaemia, and weight loss. Diabetes mellitus is present in 75-95% patients with glucagonomas [31]. Patients with somatostatinomas show a constellation of nonspecific problems. In more than 50% of these patients, a characteristic set of findings is cholelithiasis, steatorrhoea, hyperchlorhydria, and weight loss [12]. The other endocrine tumors of the pancreas are less commonly found in the body and tail.

## Diagnosis

Computed tomography (CT) scan has always been the primary imaging modality of choice in patients with pancreatic neoplasms. The advent of multi detector row helical CT (MDR CT) has reestablished CT as an important preoperative investigation. Endoscopic ultrasonography (EUS) has been also found to be useful in patients with tumors of the body and tail where a transgastric window is used for imaging and fine needle aspiration cytology (FNAC) [32]. Earlier studies had shown contrast enhanced magnetic resonance imaging (MRI) to be as accurate as helical CT in the detection and staging of pancreatic cancer [33]. However, a recent study comparing EUS, MDR CT, MRI and angiography for assessing pancreatic cancer staging and resectability demonstrated that MDR CT has the highest accuracy in assessing extent of primary tumor (73%), locoregional extension (74%), vascular invasion (83%), distant metastases (88%), tumor TNM stage (46%), and tumor resectability (83%). EUS, however, remained the modality of choice for imaging small lesions undetectable by CT as it has the highest accuracy in assessing tumor size and lymph node involvement [34]. A recent meta-analysis comparing MDR CT, MRI and ultrasonography for diagnosis and determining resectability in pancreatic cancer also proposed MDR CT (sensitivity, 91%; specificity, 85%) as the preferred method [35].

MRI, however, continues to be the modality of choice for the detection of liver metastases owing to its ability to characterize liver lesions more accurately than even contrast enhanced MDR CT [36].

Magnetic resonance cholangio-pancreatography (MRCP), despite being a very sensitive test for the detection of pancreato biliary obstruction, has low accuracy in detecting malignant features [37]. It is thus not to be routinely recommended in tumors of the body and tail.

The CT criteria for vascular invasion have been listed in Table 3 [38]. Today, with the availability of vascular reconstructions, only arterial encasement is regarded as a sign of unresectability.

**Table 3:** CT criteria for vascular invasion

Arterial embedment in tumor mass or venous obliteration
Tumor involvement exceeding one-half circumference of the vessel
Vessel wall irregularity
Vessel calibre stenosis
Teardrop sign of the superior mesenteric vein

The sensitivity and specificity of EUS-FNA in the diagnosis are said to approach 92 and 100%, respectively [39].

Laparoscopic staging has emerged as a very effective tool in detection of intraperitoneal metastasis in patients with tumors of the body and tail [40]. This is especially so in patients with suspected advanced lesions and absence of metastasis on conventional imaging. Two studies have proven the existence of unsuspected metastasis in patients with pancreatic body and tail tumors to be 36-53% [41, 42].

Regarding the tumor markers, while CA-125 may be of use in mucinous cystic neoplasms of the pancreas, the role of CA 19-9 is mainly of use when it is elevated where it helps to differentiate a malignancy from an acute inflammation. It has a sensitivity and specificity of 69-93% and 78-98%, respectively with regards to detection of pancreatic cancers [43].

In case of endocrine tumors, there are specific tests for the diagnosis of each of them the principal ones being listed in Table 4.

**Table 4:** Diagnostic tests for pancreatic endocrine tumors [12]

Tumor	Test
Insulinoma	Supervised 72hr fast, demonstrating Whipple’s triad and insulin/glucose ratio of >0.3 Selective arteriogram with intraarterial calcium injection and hepatic venous sampling Elevated c-peptide proinsulin levels
Gastrinoma	Elevated serum gastrin levels Elevated basal acid secretory rate Secretin stimulation test
Glucagonoma	Elevated glucagon levels
Somatostatinoma	Elevated fasting plasma somatostatin levels

Cystic neoplasms of the pancreatic body and tail are diagnosed by conventional imaging modalities coupled with EUS-FNA. The analysis of the cyst fluid obtained from a EUS-guided aspiration provides valuable information if analysed for biochemistry, tumor markers and not only cells as these aspirates tend to be paucicellular. In general, glycogen-rich cells are specific for serous cystadenoma, mucin-containing cells are seen in mucinous cystadenomas, and malignant cells are seen in mucinous cystadenocarcinomas [44]. Sperti et al. [45], have suggested that 18-FDG PET may be better than CT and tumor marker assays in the preoperative evaluation of patients with cystic pancreatic lesions since a positive result strongly suggests malignancy, and hence surgery, while a negative result implies a benign lesion that may be treated by limited resection or, in selected high- risk patients, with biopsy, follow-up, or both.

### TNM staging of nonendocrine pancreatic tumors [46]

Primary tumor (T)

TX:Primary tumor cannot be assessedT0:No evidence of primary tumorTis:In situ carcinomaT1:Tumor limited to the pancreas 2 cm or less in greatest dimensionT2:Tumor limited to the pancreas more than 2 cm in greatest dimensionT3:Tumor extends directly into any of the following: duodenum, bile duct, or peripancreatic tissuesT4:Tumor extends directly into any of the following: stomach, spleen, colon, or adjacent large vessels

Regional lymph nodes (N)

NX:Regional lymph nodes cannot be assessedN0:No regional lymph node metastasisN1:Regional lymph node metastasis

Distant metastasis (M)

MX:Distant metastasis cannot be assessedM0:No distant metastasisM1:Distant metastasis

AJCC stage groupings

Stage 0Tis, N0, M0Stage IT1, N0, M0 T2, N0, M0Stage IIT3, N0, M0Stage IIIT1, N1, M0 T2, N1, M0 T3, N1, M0Stage IVAT4, Any N, M0Stage IVBAny T, Any N, M1

Criteria for resectability [47]

Resectable lesions  1)No distant metastases  2)Clear fat plane around celiac and superior mesenteric arteries (SMA)  3)Patent superior mesenteric vein (SMV)/portal veinBorderline resectable lesions  1)Adrenal, colon or mesocolon, or kidney invasion  2)Preoperative evidence of biopsy positive peripancreatic lymph nodeUnresectable lesions

Distant metastasis (includes celiac and/or paraaortic lymph node metastasis); Lesions that have the above two groups of lymph nodes that are, however, in close proximity to the primary may also be regarded as borderline rather than unresectable. For body, 1) SMA/celiac/hepatic encasement; 2) SMV/portal occlusion; 3) Aortic invasion. For tail, 1) SMA/celiac encasement; 2 Rib/vertebral invasion.

## Management

Brennan et al. suggested that in patients with tumors of the body and tail of the pancreas, the approach should be aggressive and similar to the approach towards patients with tumors of the head, i.e., surgery should be contemplated as the best currently available therapeutic modality esp. for patients without known metastatic disease or major vascular invasion [48]. While it has been found at the time of surgery that approximately 35% of patients [17] have evidence of involvement of surrounding structures either by tumor infiltration or inflammatory adhesions, it is advisable that to obtain negative surgical margins, distal pancreatectomy with/without splenectomy and even en bloc resections can be resorted to. It is true that while the operative mortality following distal pancreatectomy is less than 2% [49-50], the morbidity is around 22-47% [51, 52]. This is based on the findings that the median survival after a palliative resection is much shorter than after a curative resection [53]. Shoup et al. [16], based on their extensive experience with extended resections, have concluded that patients undergoing extended resection for the adenocarcinoma of the pancreatic body and tail have long-term survival rates similar to those for patients undergoing standard resection. They also found that this group of patients had a markedly improved long-term survival compared to those who are not considered resectable because of locally advanced disease.

The prognostic factors implicated to have a poor effect on the survival following resections for tumors in the body and tail include age over 60 years, size of the tumor over 3.5cm, and an advanced stage [17]. The exact significance of an R0 resection has been debated with studies showing contradicting results [16, 48, 54]. The current opinion, though, is to be radical in obtaining an R0 margin whenever feasible.

### Closure of the pancreatic remnant, staple or suture?

The choice of closure of the pancreatic remnant has been a matter of debate with different strategies being adopted including the traditional hand-sewn closure, stapled closure, a combination of the sewn and stapled closures, application of fibrin glue or serosal patch and even the injection of prolamine [55]. Takeuchi et al. [56] found that the use of staplers to close the pancreatic remnant after a distal pancreatectomy was associated with a 0% fistula rate and was also simple and quick. A recently published meta analysis [57] that included six studies concluded that the available information showed a non-significant combined odds ratio for pancreatic fistula of 0.66 (95% confidence interval 0.35 to 1.26, *P* = 0.21) in favor of staple closure. However, a large retrospective study including 302 patients that followed this meta-analysis indicated a higher pancreatic fistula rate following the stapled closure [58]. It thus, appears that till date there is a lack of an evidence-based superiority of any one technique over the other [59].

### Role for splenectomy

While the need for spleen preservation has been propounded in benign lesions, Shoup et al. [60] have confirmed the need for splenectomy in adenocarcinomas to avoid compromising oncologic radicality.

### Role of laparoscopy

Staging laparoscopy has been shown to be a useful aid in the staging of ductal adenocarcinomas of the body and tail. In one study, 20% of patients with body and tail tumors (n = 10) were found to have liver or peritoneal metastases that was undetected on preoperative imaging [61]. In another study in which two parameters (presence of gross metastases and peritoneal fluid cytology) were assessed in 47 patients with radiologically indicated operable disease, 36% of patients (P < 0.02) were found to have inoperable disease based on these parameters [41].

For benign tumors, small islet cell tumors that have been well localized preoperatively, and for premalignant cystic lesions, laparoscopic enucleation and spleen-preserving distal pancreatectomy have been found to be feasible in selected patients [62-66]. However, for malignancies of the body and tail, further studies are required to confirm the potential benefits [67, 68].

Intraoperative ultrasonography (IOUS) is a very sensitive (93%) method to assess tumor resectability during surgery. And while it adds little time and no morbidity to the operation, it aids intra-operative decision-making [69].

### Role of modified appleby in distal pancreatic tumors

The modified Appleby procedure consists of ligation of the celiac artery at its start point, common hepatic artery, along with distal pancreatectomy and removal of celiac plexus and ganglions, as well as, retroperitoneal tissues. The procedure is based on the presence of collateral circulation between the superior mesenteric artery and the hepatobiliary system by way of an intact pancreaticoduodenal arcade. This procedure has been indicated in locally advanced lesions involving the celiac axis without invasion of the head of the pancreas, and proper hepatic artery and superior mesenteric arteries [70]. There should be clear pulsations of the proper hepatic artery 1-2 min after occlusion of the common hepatic artery. The advantages of this radical procedure have been the ability to salvage locally advanced tumors, while decreasing postoperative pain due to the removal of the celiac plexus. While the results remain unsatisfactory as this procedure requires a tedious vascular workup, patients with celiac axis involvement secondary to central pancreatic tumors need not be regarded as unresectable [71].

### Surgical management of cystic pancreatic tumors

The currently accepted guidelines are that for SCAs, an organ preserving resection should be carried out while for mucinous tumors, a more radical resection is advised [72]. A segmental or distal pancreatectomy with preservation of the spleen where possible is recommended [73-78]. Distal pancreatectomy with splenectomy should be carried out in patients with MCAC [79] In the case of IPMN, where the tendency is for the tumor to grow along the ducts rather than radially into the parenchyma, the resection margins must be examined by frozen section intra operatively to confirm the clearance of the margins. The overall 5-year survival nears 100% for SCA and even MCA where the resection margins are clear and there is no evidence of transmural invasion [20, 80]. Even in IPMNs containing carcinoma, 5-year survival is over 50% [81].

Walsh et al. [82] have even suggested that with the increasing incidence of ‘incidentally’ detected asymptomatic pancreatic cysts, if a mucinous neoplasm can be excluded with confidence, an EUS guided aspiration can be done and the patient followed up clinically and with interval imaging.

### Surgical management of endocrine tumors

The basic principles of surgery in these tumors are to avoid blind resections. Surgery should be considered with caution in these patients, as it is wise to adopt the adage that the treatment should not be more aggressive and symptom producing than either the lesion or the manifestations of the disease. While in localized lesions a radical surgery can be performed, in large lesions, a debulking surgery may be resorted to. The main surgeries done for these tumors are: enucleation for benign tumors like insulinomas and gastrinomas, and radical resection for most others that have a higher likelihood of being malignant [83].

Total gastrectomy, which was considered an integral part of gastrinoma treatment, is now regarded as obsolete except in few indolent cases due to the availability of proton pump inhibitors.

Every attempt should be made to localise the tumor preoperatively by use of imaging modalities. In the event that the tumor is not localised, the patient should be explored by a generous incision with a thorough intraoperative search for the lesions after adequate mobilisation of the body and tail of the pancreas. This is possible after dividing the peritoneal attachments along the superior and inferior border of the pancreas. By this maneuver, the entire pancreas can be felt between the fingers enabling detection of the lesions that, most often, are very small. Liberal use of intraoperative ultrasound is encouraged.

Evidence of multicentricity, lymph node metastasis, and hepatic metastasis should be sought at the time of surgery by way of sampling. In which case en bloc lymphadenectomy and hepatic resections should be resorted to when feasible [84-90].

### Role of segmental resections

In the recent years, there have been increasing reports of organ sparing (central pancreatectomy) resections being undertaken for lesions in the body and tail [91-93]. The advantage of such resections is the lowered incidence of complications such as postoperative exocrine-endocrine dysfunction and post-splenectomy sepsis [91]. While this procedure is attractive, the reported pancreatic fistula rates after a central pancreatectomy have been reported to be as high as 51 - 63% [91, 94].

Another issue with central pancreatectomy is the risk of inadequate resections in the case of malignant tumors [94]. Hence it is indicated at the present time mainly for benign / low-grade malignant lesions or metastases to the neck or proximal body of the pancreas. The use of such resections for ductal adenocarcinomas is not indicated owing to the entirely different and aggressive tumor biology [95].

### Role of lymphadenectomy

The role for extended lymphadenectomy in tumors of the body and tail has always been promulgated by Ozaki [96] and Shoup [16] on the premise that surgery is the only chance for cure. While assessing the prognostic factors affecting survival in pancreatic body and tail tumors, Shimada et al. [97], found that unlike tumors in the head where the portal vein presents the surgeon with a daunting vascular reconstruction, the invasion of the splenic vein is surgically easier to handle. The application of extended lymphadenectomy has provided the ability to precisely stage the tumor while ensuring a clear operative margin. Shimada concluded that despite his experience with no increased operative mortality with extended lymphadenectomy, further randomised trials are needed to establish it as a standard procedure for tumors of the body and tail.

## Complications after distal pancreatectomy

The complications after surgery for the pancreatic body and tail tumors are classified into major and minor [48]. The major complications encountered include infections/abscess, pancreatic fistula, sepsis, small bowel fistula, upper GI bleed, and small bowel obstruction. The minor complications that can be encountered include fever, nausea/vomiting, tachycardia, edema, ileus, and respiratory complications.

## Role of chemotherapy and radiotherapy

### Adjuvant therapy in resectable disease

The use of gemcitabine vs 5FU, before, and after chemoradiation in resectable pancreatic adenocarcinomas [98] failed to show any improvement in survival in patients with tumors of the body and tail though patients who received gemcitabine did show a better 3-year survival.

### In locally advanced disease

A randomized phase III study by the FFCD-SFRO [99] compared the use of initial chemoradiation followed by gemcitabine with gemcitabine alone in patients with locally advanced pancreatic cancer. The primary end point was overall survival (OS). The toxicity in the chemoradiotherapy arm probably led to the decreased use of maintenance gemcitabline in that arm. This led to a poor overall survival for the chemoradiotherapy arm leading the author to conclude that in locally advanced pancreatic cancer, gemcitabine should be the only drug used. Another study by Wilkowski et al. [100] has concluded that gemcitabine and 5FU can be safely combined with external beam radiotherapy to help achieve a secondary resection.

The studies examining the role of chemoradiotherapy in advanced pancreatic carcinoma are summarized in Table 5 (Saif MW – Online CME “Treatment of pancreatic cancer”) [100-104].

**Table 5:** Chemotherapy in advanced / metastatic pancreatic adenocarcinoma (Saif MW – Online CME “Treatment of pancreatic cancer) [101-104]

Study	Gemcitabine regimen	Median Survival (months)	1-year survival
Burris et al [102]	30-minute infusion	5.7	18%
Tempero et al [103]	Fixed dose rate	7.8	24%
Louvet et al [104]	Gemcitabine and Oxaliplatin	9.2	36%

### Role of neoadjuvant chemotherapy along with radiotherapy in resectable disease

Two recent phase II trials explored the possibility of this intervention. Talamonti et al. [105] found that the benefit of the use of gemcitabine along with radiotherapy included a survival ranging up to 11 months but the toxicities and the low number of patients who could eventually undergo an R0 resection has not permitted the use of this routinely outside the confines of clinical trials. Mornex et al. [106] proposed that this pre-operative scheme is feasible, does not prevent successful surgery, and must be tested on a Phase III setting.

In cystic pancreatic tumors, as well, the role of chemoradiation requires more studies to prove it’s efficacy as the currently available evidence is based on experimental experience with few patients and information acquired from case reports. The indications cited in available literature include evidence of tissue invasiveness [107-109] in the pathological specimen, liver metastasis (chemoembolization) [110], unresectable tumor (radiotherapy) [111], large tumors (neoadjuvant chemotherapy to downsize the tumor for surgery) [112], and with aneuploid neoplasms [113]. Sarr et al. have suggested a role for adjuvant treatment even in the absence of lymph node metastasis [4].

In endocrine tumors, the role for adjuvant treatment is immense. The various therapies used include biological modifiers, 1) Somatostatin Analogues: This group of drugs has been used in patients wherein the complete surgical removal of the tumor is not possible, or there is the presence of extensive liver metastases, wide-spread local invasion or rapid growth. The introduction of analogues with sustained release from depot injections, which can be given every 2 - 4 weeks, have made dosaging more convenient (Sandostatin LAR – monthly, Lanreotide – fortnightly, and lanreotide autogel – monthly). These drugs, lanreotide (fortnightly injection), Sandostatin LAR (monthly), and Lanreotide Autogel (also monthly), have been associated with significant improvement in the quality of life of patients [114-116]; 2) Targeted radionuclide therapy: The primary indications have been as palliation in patients who are inoperable, metastatic and progressive tumors with avid uptake of ^123^I-MIBG or ^111^In-octreotide at all known tumor sites on diagnostic imaging. Although no randomized controlled trials have been performed, current data is encouraging in terms of tumor stabilization. The isotopes used include ^123^I-MIBG, ^90^Y-octreotide for ^111^In-octreotate, and ^90^Y-lanreotide for ^111^In-lanreotide and ^177^lutetium. For ^131^I-MIBG therapy, symptom control is up to 80% with a 5-year survival rate of 60% [117-118]. For ^90^Y-octreotide therapy, the majority of patients achieve tumor stabilization although significant tumor regression is unusual [119-121]; 3) Interferons: Results utilizing interferon indicate a mean biochemical response rate of 40-60%, symptomatic improvement in 40-70%, and tumor shrinkage in about 10-15% [122-124].

### Chemotherapy

The role of chemotherapy for endocrine pancreatic tumors is uncertain. Recommendations for well-differentiated endocrine carcinomas consist of combinations including permutations or streptozotocin, epirubicin, dacarbazine, 5-fluorouracil, and adriamycin, and response rates vary between 40% and 70% [125, 126]. The unpredictable response rates debilitating side effects in insulinomas, carcinoids, and VIPomas limit their use in these tumors. Response rates of > 70% to cisplatin and etoposide combination chemotherapy have been seen in the poorly differentiated and anaplastic endocrine pancreatic tumors [127, 128].

### Emerging therapies

Targeted therapy in exocrine pancreatic tumors, an approach to combating pancreatic tumors, has emerged owing to the better understanding of the molecular biology of pancreatic cancer, and the envisaged marginal benefits that can be obtained even with the best chemotherapeutic regimens. The main receptor that is being targeted today is the ErbB receptors viz., ErbB1 – EGFR, and ErbB2 – Her2. Cetuximab, a humanized monoclonal antibody to ErbB1 [129] and Trastuzumab, an antibody to Her2 [130] are currently being used in combination with gemcitabine. Trials are on to evaluate the role of matrix metalloproteinases [131].

Novel somatostatin radionuclide therapies are currently in development. ^188^Re-octreotide may be substituted for ^99m^Tc-EDTA-HYNIC-octreotide [16].^ 177^Lu-octreotate therapy has been recently introduced [132]. Other novel agents involving cell signaling transduction blockers (e.g. Gifitinib), which affect tyrosine kinase and a variety of angiogenesis inhibitors are under investigation.

## Prognosis

The 5-year survival rate of pancreatic body and tail tumors after surgical resection ranges from 0-25%, and the median survival time is 10-15.9 months [16, 48, 133-135]. This post resection survival rate, however, does not differ depending on whether the tumor is in the head, body, or tail [48].
